# Telmisartan Activates Endothelial Nitric Oxide Synthase via Ser1177 Phosphorylation in Vascular Endothelial Cells

**DOI:** 10.1371/journal.pone.0096948

**Published:** 2014-05-14

**Authors:** Masahiro Myojo, Daisuke Nagata, Daishi Fujita, Arihiro Kiyosue, Masao Takahashi, Hiroshi Satonaka, Yoshiyuki Morishita, Tetsu Akimoto, Ryozo Nagai, Issei Komuro, Yasunobu Hirata

**Affiliations:** 1 Department of Cardiovascular Medicine, The University of Tokyo, Graduate school of Medicine, Tokyo, Japan; 2 Department of Nephrology, Jichi Medical University, Tochigi, Japan; 3 Department of Cardiology and Nephrology, Dokkyo Medical University, Tochigi, Japan; 4 Jichi Medical University, Tochigi, Japan; 5 The Tokyo Teishin Hospital, Tokyo, Japan; Osaka University Graduate School of Medicine, Japan

## Abstract

Because endothelial nitric oxide synthase (eNOS) has anti-inflammatory and anti-arteriosclerotic functions, it has been recognized as one of the key molecules essential for the homeostatic control of blood vessels other than relaxation of vascular tone. Here, we examined whether telmisartan modulates eNOS function through its pleiotropic effect. Administration of telmisartan to mice significantly increased the phosphorylation level of eNOS (Ser1177) in the aortic endothelium, but administration of valsartan had no effect. Similarly, telmisartan treatment of human umbilical vein endothelial cells significantly increased the phosphorylation levels of AMP-activated protein kinase (Thr172) and eNOS and the concentration of intracellular guanosine 3′,5′-cyclic monophosphate (cGMP). Furthermore, pretreatment with a p38 mitogen-activated protein kinase (p38 MAPK) inhibitor suppressed the increased phosphorylation level of eNOS and intracellular cGMP concentration. These data show that telmisartan increases eNOS activity through Ser1177 phosphorylation in vascular endothelial cells mainly via p38 MAPK signaling.

## Introduction

The renin-angiotensin system (RAS) is one of the most pivotal mechanisms that modulate blood pressure. Furthermore, it is known to be the upstream cause of various cardiovascular diseases such as atherosclerosis, hypertension, ventricular hypertrophy, myocardial infarction, and heart failure [1]. Angiotensin-converting enzyme (ACE) inhibitors and angiotensin II receptor blockers (ARBs) are frequently prescribed for the treatment of hypertension. They also reduce the risk of renal dysfunction and the incidence of cardiovascular diseases [1], [2], [3], [4], [5]. The effects of RAS inhibitors are mostly common within the same drug class, but some effects are reported to be drug-specific. Among ARBs, the unique function of telmisartan in the adipose tissue is mediated through peroxisome proliferator-activated receptor (PPAR)γ activation. However, the mechanism of the vascular protective effect of telmisartan is not fully understood. Both endothelial nitric oxide synthase (eNOS) and AMP-activated protein kinase (AMPK) have been suggested to play a role in the vascular endothelium to protect against the deteriorating effects of oxidative stress. Recently, post-translational phosphorylation of eNOS by kinases is considered to play an important role in regulation of eNOS activity [6], [7]. Five phosphorylation sites in eNOS have been identified and Ser1177 is considered to be the most important phosphorylation site of its enzyme activity. AMPK plays a protective role in vascular endothelial cells through cellular autophagy and by suppression of apoptosis [8]. It has also been reported that AMPK is essential for angiogenesis in response to hypoxic stress [9].

In this study, we examined the vascular protective effect of ARBs with relation to their ability to activate eNOS and AMPK. Additionally, we examined which signaling pathway plays a pivotal role in eNOS activation by telmisartan in human umbilical vein endothelial cells (HUVECs).

## Materials and Methods

### Materials

Antibodies against phospho-eNOS (Ser1177), phospho-AMPKα (Thr172), AMPKα, phospho-Akt (Ser473), phospho-p38 mitogen-activated protein kinase (p38 MAPK) (Thr180/Tyr182), p38 MAPK and cAMP response element binding protein (CREB) were purchased from Cell Signaling Technology (Danvers, MA). Antibodies against phospho-CREB (Ser133) and eNOS (NOS3) were purchased from Santa Cruz Biotechnology (Santa Cruz, CA). An anti-phospho-acetyl CoA carboxylase (ACC) (Ser79) antibody was purchased from Millipore (Billerica, MA). An anti-myc tag antibody was purchased from Upstate Biotechnology (Lake Placid, NY) and an anti-HA tag antibody was purchased from Roche (Basel, Switzerland).

Wortmannin, LY294002, and KT5720 were purchased from Cayman Chemical Company (Ann Arbor, MI). H89 and GSK3787 were purchased from Tocris Bioscience (Bristol, UK). GW9662 was purchased from Wako Pure Chemical (Osaka, Japan). SB202190 was purchased from Calbiochem (Darmstadt, Germany).

Valsartan and irbesartan were purchased from Vijayasri Chemicals (Andhra Pradesh, India). Telmisartan was provided by Boehringer Ingelheim (Ingelheim, Germany).

### Adenovirus

The replication-defective adenoviral vector expressing dominant-negative AMPK was identical to that used in a previous report [10]. This vector overexpresses the rat AMPK α2-subunit in which lysine45 has been mutated to arginine and is fused in-frame with the c-Myc epitope tag. An adenoviral vector expressing green fluorescent protein (GFP) was obtained from Qbiogene (Illkirch, France) and used to assess transduction efficiency. An adenoviral vector expressing dominant negative p38 MAPK fused with a HA epitope tag was a generous gift from Dr. Mitsuyama and this was used in a previous report [11].

### Cell culture and adenovirus infection

HUVECs were purchased from Kurabo (Osaka, Japan) and cultured in HuMedia EG2 (Kurabo). In some experiments, HUVECs were transduced with the indicated replication-defective adenoviral vectors at a multiplicity of infection (MOI) of 50 for 1 day. The medium was then changed to HuMedia basic medium (EB2) with 0.2% FBS to reduce stimulation by serum mitogens. In all experiments, HUVECs were used at passage 7 or less.

### Drug treatments

After incubation in EB2 containing 0.2% FBS for 1 day, the cells were treated with inhibitors, antagonists and ARBs diluted in dimethyl sulfoxide (DMSO).

### Western blot analysis

Western blotting was carried out as described previously [12], [13]. An ECL-PLUS Western Blotting Detection kit (GE Healthcare, Piscataway, NJ) was used for detection. The density of the bands was quantified using the Scion Image program. Each experiment was repeated 3–4 times.

### Immunohistochemical staining of mouse aortic endothelium

The animal care and use procedures were approved by the Animal Care Committee of the University of Tokyo (Permit Number: P09-015). Male C57 BL mice were purchased from Oriental Yeast (Tokyo, Japan) and fed standard laboratory chow (CLEA Japan, Tokyo, Japan). After drug treatment for 7 days, the mice were sacrificed to extract the descending aortas. The tissues were fixed in 4% paraformaldehyde, dehydrated, and then embedded in paraffin. The paraffin-embedded tissues were cut into cross sections (2 µm). The sections were deparaffinized and rehydrated for immunohistochemistry. Tissue sections were incubated with rabbit anti-phospho eNOS (Ser1177) or -eNOS antibodies diluted at 1:200, followed by a biotinylated anti-rabbit secondary antibody and then horseradish peroxidase-labeled streptavidin, according to the manufacturer's instructions (DAKO, Copenhagen, Denmark). Staining intensities were measured in 30 randomly selected fields of the endothelium and media by Scion Image program. The intensity ratio of the endothelium to media was then compared statistically.

### cGMP assay

To evaluate nitric oxide (NO) output from HUVECs, the intracellular cGMP concentration was measured using a cGMP enzyme immunoassay system (R&D Systems, Minneapolis, MN) according to the manufacturer's instructions and our previous reports [8], [13]. After incubation in low serum medium for 8 h, the cells were incubated in serum-free medium containing telmisartan or other reagents for 4 h. Each experiment was performed using 4 independent samples for 3 separate experiments.

### Statistical analysis

Values are expressed as the mean ± S.E. Statistical comparisons were performed using analysis of variance, and the Tukey-Kramer method was used for comparisons of means between groups. A P-value of less than 0.05 was considered statistically significant.

## Results

### Telmisartan phosphorylates eNOS Ser1177 and AMPKα Thr172 in HUVECs

**Figure 1:**
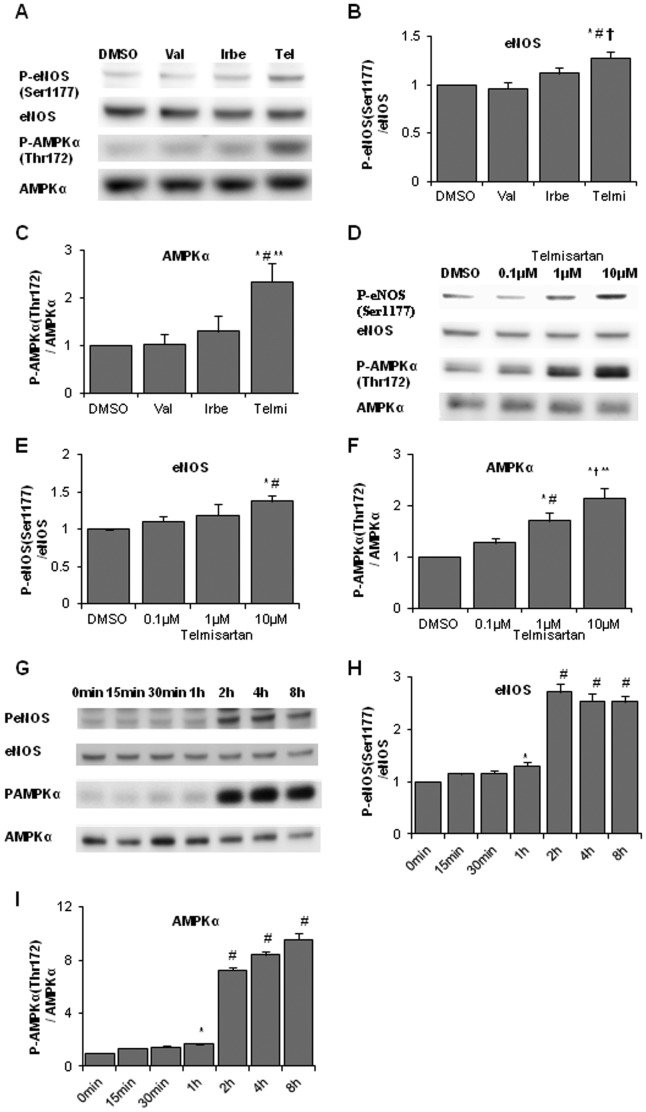
ARB induces eNOS and AMPK phosphorylation in HUVECs. **A**. HUVECs were incubated with the vehicle (DMSO), valsartan (10 µM), irbesartan (10 µM), or telmisartan (10 µM) for 2 h. Representative data are shown. **B**. Relative phosphorylation levels of eNOS calculated as the ratio of phospho-eNOS to total eNOS. Bar graph shows the mean ± SEM of 4 independent experiments. **C**. Relative phosphorylation levels of AMPK calculated as the ratio of phospho-AMPK to total AMPK. Bar graph shows the mean ± SEM of 4 independent experiments. Val, valsartan; Irbe, irbesartan; Telmi, telmisartan. **B**, **C**: *, p < 0.01 versus control; #, p < 0.01 versus Val; †, p < 0.05 versus Irbe; **, p < 0.01 versus Irbe. **D**. HUVECs were incubated with the vehicle (DMSO) or telmisartan (0.1, 1, and 10 µM) for 2 h. A representative blot is shown. **E**. Relative phosphorylation levels of eNOS were quantified as described in **B**. Bar graph shows the mean ± SEM of 4 independent experiments. **F**. Relative phosphorylation levels of AMPK were quantified as described in **C**. Bar graph shows the mean ± SEM of 4 independent experiments. **E**, **F**: *, p < 0.01 versus control (DMSO); #, p < 0.05 versus 0.1 µM telmisartan; †, p < 0.01 versus 0.1 µM telmisartan; **, p < 0.05 versus 1 µM telmisartan. **G**. HUVECs were incubated with telmisartan (10 µM) for 15 min to 8 h. **H**. Relative phosphorylation levels of eNOS calculated as the ratio of phospho-eNOS to total eNOS. Bar graph shows the mean ± SEM of 4 independent experiments. **I**. Relative phosphorylation levels of AMPK calculated as the ratio of phospho-AMPK to total AMPK. Bar graph shows the mean ± SEM of 4 independent experiments. **H**, **I**: *, p < 0.05 versus control (0 min); #, p < 0.01 versus control.

Western blot analyses were performed to compare the phosphorylation levels of eNOS Ser1177 and AMPKα Thr172 in HUVECs stimulated by three different ARBs, valsartan, irbesartan, and telmisartan, at 10 µM. Significant increases were observed in eNOS and AMPKα phosphorylation levels following treatment with telmisartan, but not the vehicle, valsartan, or irbesartan (Fig. 1A–C).

We also examined concentration-dependent differences in eNOS and AMPK phosphorylation levels induced by telmisartan. After 24 h of incubation in low serum, HUVECs were incubated with DMSO, telmisartan (0.1, 1, or 10 µM) for 2 h followed by protein extraction. In telmisartan-treated cells, phosphorylation levels of AMPK Thr172 and eNOS Ser1177 increased in a concentration-dependent manner (Fig. 1D–F). In valsartan- and irbesartan-treated cells, we found no significant upregulation of phosphorylation levels (data not shown).

Next, we examined time-dependent increases in eNOS and AMPK phosphorylation by telmisartan. HUVECs were incubated with telmisartan for 15 min to 8 h followed by protein extraction for western blot analyses. The ratio of phospho-eNOS (Ser1177)/eNOS significantly increased at 1 h and reached the maximum at 2 h. Furthermore, the ratio of phospho-AMPKα (Thr172)/AMPKα significantly increased from 1 h and reached the maximum at 8 h (Fig. 1 G–I).

### Oral administration of telmisartan increases eNOS phosphorylation in the mouse endothelium of the descending aorta

**Figure 2:**
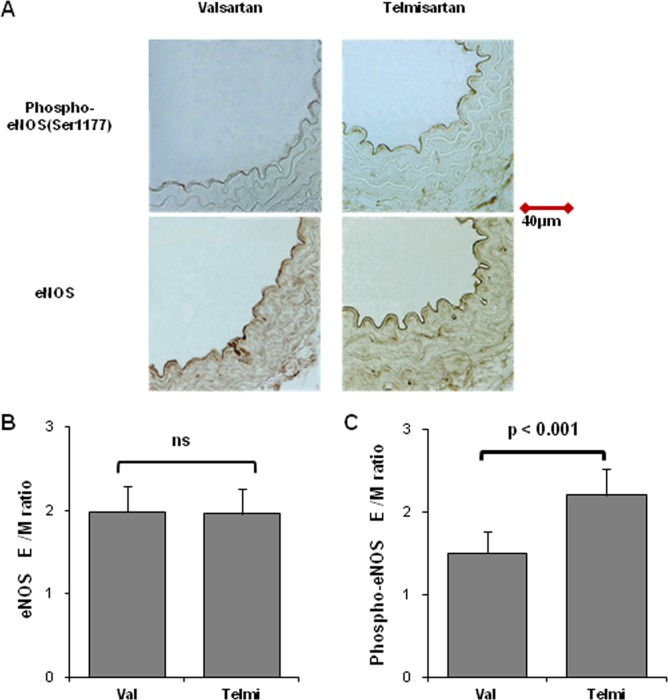
Immunohistochemical staining of aortic endothelium treated with valsartan and telmisartan. **A**. Mouse descending aortas after oral administration with valsartan (5 mg⋅kg^−1^⋅day^−1^) or telmisartan (2.5 mg⋅kg^−1^⋅day^−1^) for 7 days were immunostained using anti-phospho eNOS (Ser1177) and anti-eNOS antibodies. **B**, **C**. Staining intensities were measured in 30 randomly selected field of endothelium (E) and media (M). The intensity ratios of the endothelium to media were compared statistically.

Next, we examined whether oral administration of telmisartan could increase eNOS phosphorylation levels in vivo, as was observed in cultured HUVECs. C57 BL6 mice were treated with methyl cellulose (vehicle), valsartan (5 mg⋅kg^−1^⋅day^−1^) or telmisartan (2.5 mg⋅kg^−1^⋅day^−1^) for 7 days. At 4 h after the last administration, the mice were sacrificed and the aorta was isolated. Immunohistochemical analysis showed similar staining intensities of anti-eNOS antibody in valsartan and telmisartan groups, but the staining intensity of an anti-phospho-eNOS (Ser1177) antibody in the telmisartan group was almost 1.5 times higher than that in the valsartan group (Fig. 2A–C).

### Pretreatment with high concentrations of valsartan does not inhibit eNOS and AMPK phosphorylation by telmisartan in HUVECs

**Figure 3:**
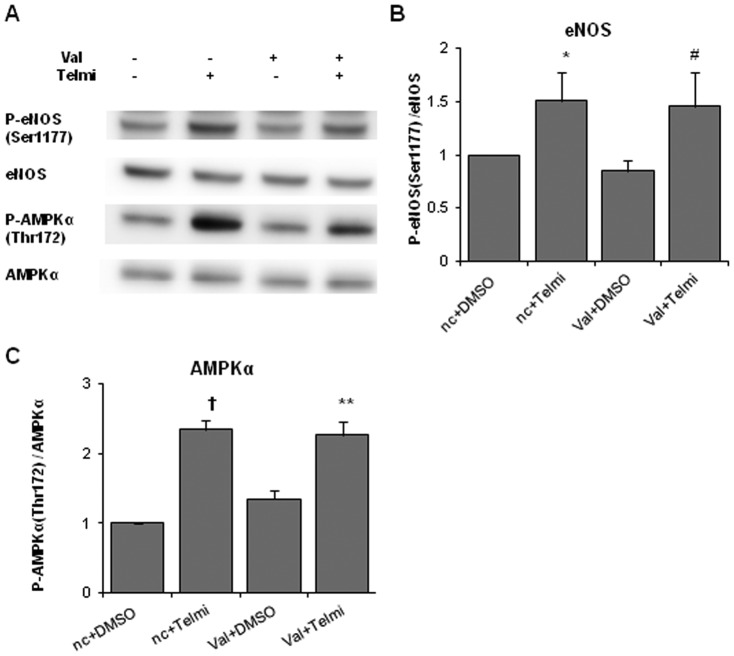
AT1 inhibition does not inhibit eNOS or AMPK phosphorylation induced by telmisartan in HUVECs. **A**. HUVECs were incubated with the vehicle (DMSO) or telmisartan for 2 h under the conditions, in which the AT1R binding pocket was blocked by pretreatment with high concentrations of valsartan (200 µM) for 30 min. Representative immunoblots of eNOS and AMPK are shown. **B**. Relative phosphorylation levels of eNOS were calculated as the band intensity ratio of phospho-eNOS to total eNOS. Bar graph shows the mean ± SEM of 4 independent experiments. **C**. Relative phosphorylation levels of AMPK were calculated as the band intensity ratio of phospho-AMPK to total AMPK. Bar graph shows the mean ± SEM of 4 independent experiments. nc, negative control. *, p < 0.05 versus control (nc+DMSO); #, p < 0.05 versus Val+DMSO; †, p < 0.01 versus control; **, p < 0.01 versus Val+DMSO.

To examine whether telmisartan phosphorylates eNOS and AMPK through binding to AT1R, HUVECs were incubated with telmisartan for 2 h under a condition in which the AT1R binding pocket was blocked by 30 min of pretreatment with high concentrations of valsartan (200 µM). The results of western blot analyses showed that the levels of eNOS and AMPK phosphorylation induced by telmisartan were independent of pretreatment with valsartan (Fig. 3A, B).

### Inhibition of either phosphatidylinositol-3 kinase (PI3K) or protein kinase A (PKA) does not inhibit telmisartan-induced eNOS phosphorylation in HUVECs

**Figure 4:**
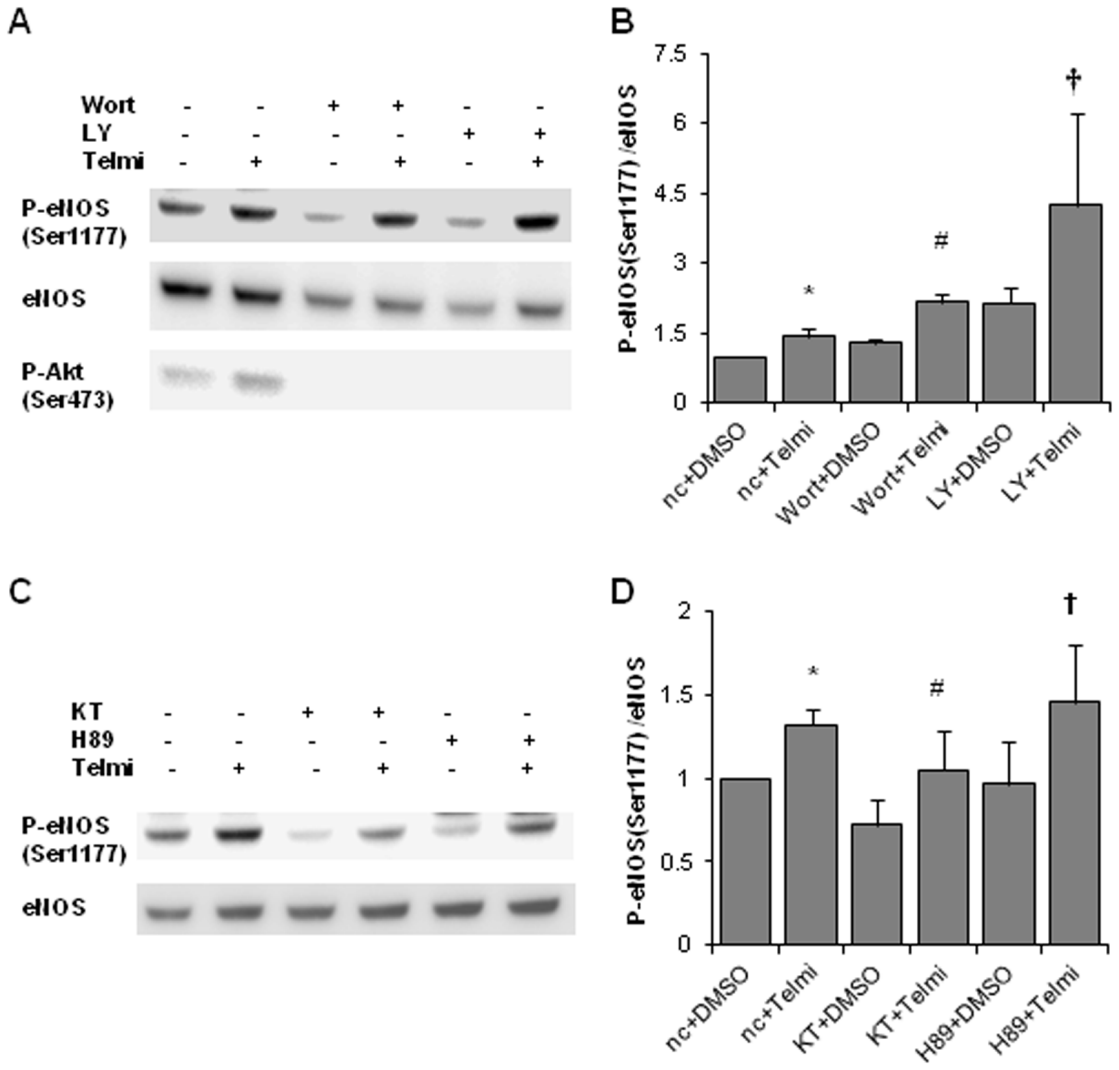
Telmisartan-induced eNOS phosphorylation levels after pretreatment with PI3K or PKA inhibitors. **A**. HUVECs were incubated with the vehicle (DMSO) or telmisartan for 2 h after pretreatment with PI3K inhibitors wortmannin (1 µM) or LY294002 (50 µM) for 30 min. **B**. Relative phosphorylation levels of eNOS calculated as the ratio of phospho-eNOS to total eNOS. Bar graph shows the mean ± SEM of 4 independent experiments. Wort, wortmannin; LY, LY294002. *, p < 0.05 versus control (nc+DMSO); #, p < 0.05 versus Wort+DMSO; †, p < 0.05 versus LY+DMSO. **C**. HUVECs were incubated with the vehicle (DMSO) or telmisartan for 2 h after pretreatment with PKA inhibitors KT5720 (5 µM) or H89 (10 µM) for 30 min. **D**. Relative phosphorylation levels of eNOS calculated as the ratio of phospho-eNOS to total eNOS. Bar graph shows the mean ± SEM of 4 independent experiments. KT, KT5720. *, p < 0.05 versus control (nc+DMSO); #, p < 0.05 versus KT+DMSO; †, p < 0.05 versus H89+DMSO.

We evaluated the involvement of PI3K-Akt in telmisartan-induced eNOS phosphorylation. Pretreatment with PI3K inhibitors wortmannin (1 µM) or LY294002 (50 µM) for 30 min did not suppress eNOS phosphorylation induced by telmisartan, suggesting that PI3K signaling is independent of such eNOS phosphorylation (Fig. 4A, B). We next evaluated the involvement of PKA in telmisartan-induced eNOS phosphorylation by pretreatment with PKA inhibitors KT5720 (5 µM) and H89 (10 µM). Neither inhibitor suppressed telmisartan-induced eNOS phosphorylation, suggesting that PKA is not involved in such phosphorylation (Fig. 4C, D).

### PPAR inhibition does not inhibit telmisartan-induced eNOS phosphorylation

**Figure 5:**
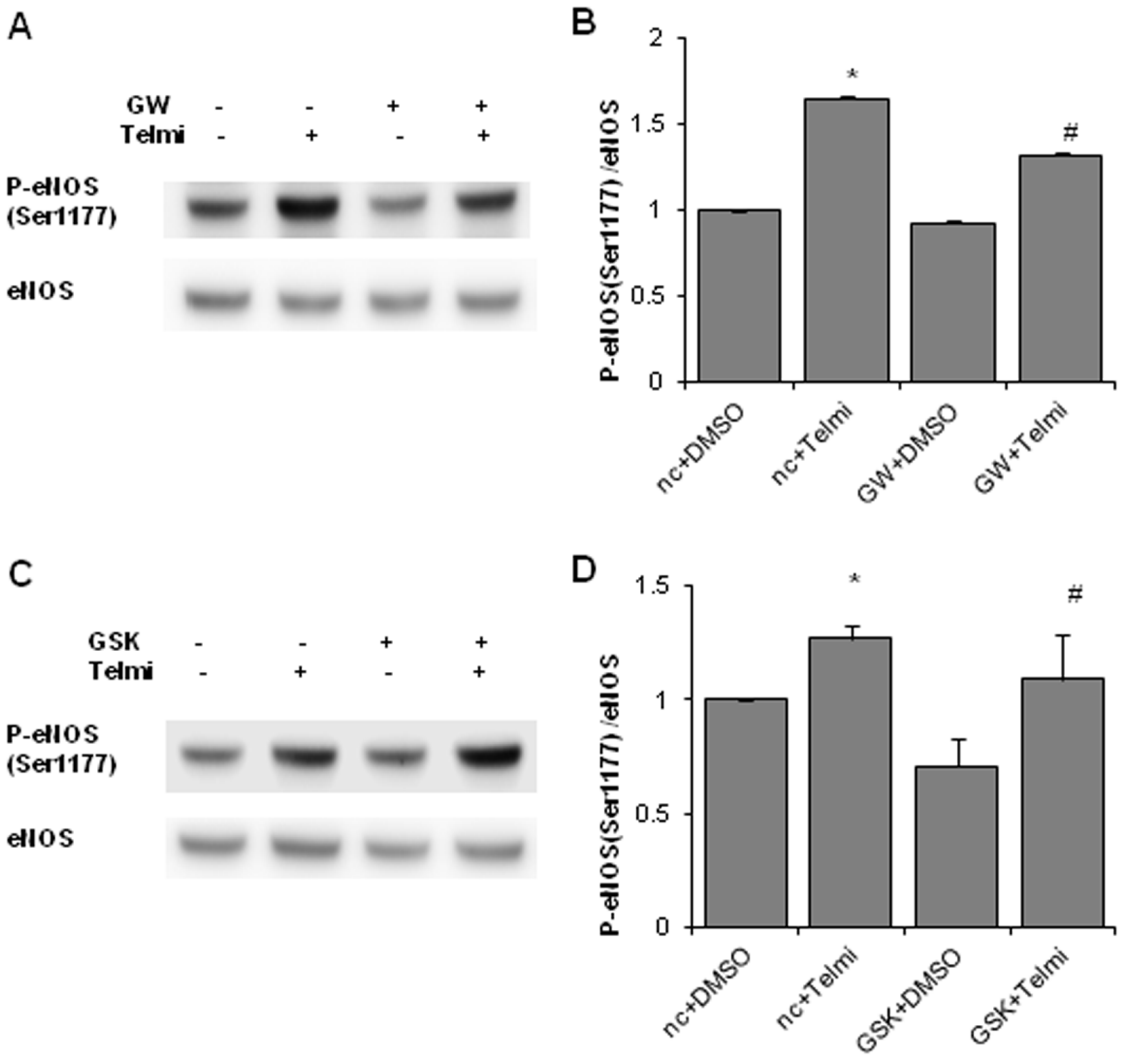
Telmisartan-induced eNOS phosphorylation levels after pretreatment with PPARγ or PPARβ/δ inhibitors. **A**. HUVECs were incubated with the vehicle (DMSO) or telmisartan for 2 h after pretreatment with the PPARγ inhibitor GW9662 (10 µM) for 30 min. **B**. Relative phosphorylation levels of eNOS calculated as the ratio of phospho-eNOS to total eNOS. Bar graph shows the mean ± SEM of 4 independent experiments. GW, GW9662. *, p < 0.01 versus control (nc+DMSO); #, p < 0.01 versus GW+DMSO. **C**. HUVECs were incubated with the vehicle (DMSO) or telmisartan for 2 h after pretreatment with the PPARβ/δ inhibitor GSK3787 (10 µM) for 30 min. **D**. Relative phosphorylation levels of eNOS calculated as the ratio of phospho-eNOS to total eNOS. Bar graph shows the mean ± SEM of 4 independent experiments. GSK, GSK3787. *, p < 0.05 versus control (nc+DMSO); #, p < 0.05 versus GSK+DMSO.

We next evaluated the involvement of PPARγ and PPARβ/δ in telmisartan-induced eNOS phosphorylation. eNOS Ser1177 phosphorylation induced by telmisartan was unchanged by pretreatment with the PPARγ antagonist GW9662 (10 µM) (Fig. 5 A, B) or PPARβ/δ antagonist GSK 3787 (10 µM) for 30 min (Fig. 5C, D).

### AMPK inhibition does not inhibit eNOS phosphorylation by telmisartan in HUVECs

**Figure 6:**
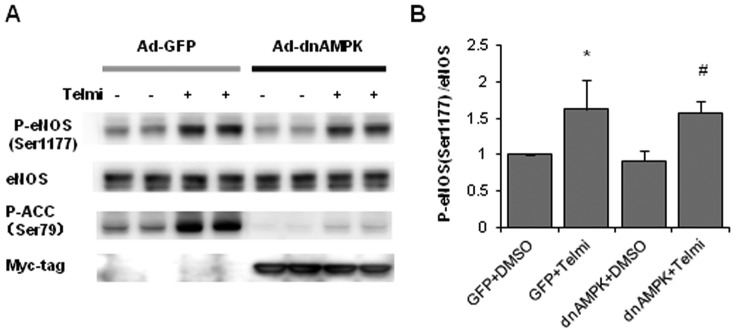
AMPK inhibition does not inhibit eNOS phosphorylation induced by telmisartan in HUVECs. **A**. HUVECs were incubated with the vehicle (DMSO) or telmisartan for 2 h after overexpression of dominant-negative AMPK (dnAMPK) or GFP by adenoviral vectors. Phosphorylation levels of ACC indicate the activity of AMPK. Myc-tag expression was checked to verify overexpression of dnAMPK. **B**. Relative phosphorylation levels of eNOS calculated as the ratio of phospho-eNOS to total eNOS. Bar graph shows the mean ± SEM of 4 independent experiments. *, p < 0.05 versus control (GFP+DMSO); #, p < 0.05 versus dnAMPK+DMSO.

Next, we evaluated the involvement of AMPK in telmisartan-induced eNOS phosphorylation. Overexpression of dominant-negative AMPK by an adenoviral vector did not suppress telmisartan-induced eNOS phosphorylation (Fig. 6A,B), suggesting that AMPK is not necessary for eNOS Ser1177 phosphorylation induced by telmisartan.

### Involvement of p38 MAPK activation in telmisartan-induced eNOS phosphorylation in HUVECs

**Figure 7:**
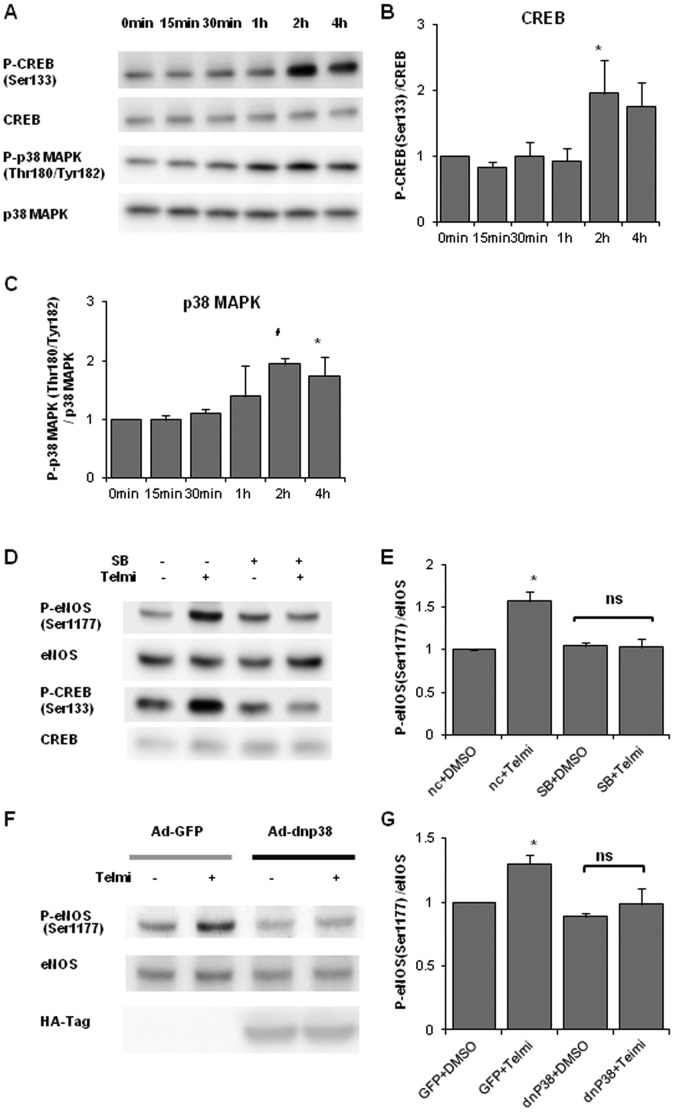
Telmisartan induces p38 MAPK and CREB phosphorylation in HUVECs. **A**. HUVECs were incubated with telmisartan for 15 min to 4 h to evaluate the phosphorylation levels of p38MAPK Thr180/Tyr182 and CREB Ser133. **B**. Relative phosphorylation levels of CREB calculated as the ratio of phospho-CREB (Ser133) to total CREB. Bar graph shows the mean ± SEM of 4 independent experiments. **C**. Relative phosphorylation levels of p38 MAPK calculated as the ratio of phospho-p38 MAPK to total p38 MAPK. Bar graph shows the mean ± SEM of 4 independent experiments. **B**, **C**: *, p < 0.05 versus control (0 min); #, p < 0.01 versus control. **D**. HUVECs were incubated with the vehicle (DMSO) or telmisartan for 2 h under the condition of p38 MAPK inhibition by pretreatment with SB202190 (10 µM) for 30 min. **E**. Relative phosphorylation levels of eNOS calculated as the ratio of phospho-eNOS to total eNOS. Bar graph shows the mean ± SEM of 4 independent experiments. SB, SB202190. *, p < 0.01 versus control (nc+DMSO). **F**. HUVECs were incubated with the vehicle (DMSO) or telmisartan for 2 h after overexpression of dominant-negative p38 MAPK (dn p38 MAPK) or GFP by adenoviral vectors. HA-tag expression was checked to verify overexpression of dn p38 MAPK. **G**. Relative phosphorylation levels of eNOS calculated as the ratio of phospho-eNOS to total eNOS. Bar graph shows the mean ± SEM of 4 independent experiments. *, p < 0.05 versus control (GFP+DMSO).

We evaluated the phosphorylation levels of p38 MAPK and its downstream target CREB in telmisartan-treated HUVECs. CREB Ser133 is a representative phosphorylation site of p38 MAPK. HUVECs were incubated with telmisartan for 15 min to 4 h followed by protein extraction. The band intensity ratios, phospho-CREB Ser133/CREB, and phospho-p38 MAPK Thr180/Tyr182/p38 MAPK, were significantly increased by telmisartan treatment and it reached a maximum at 2 h (Fig. 7A–C).

We next evaluated the involvement of p38 MAPK in telmisartan-induced eNOS phosphorylation by using the p38 MAPK inhibitor SB202190 (10 µM). After SB202190 pretreatment, telmisartan-induced eNOS phosphorylation was suppressed to basal levels (Fig. 7D, E). We also investigated whether telmisartan-induced phosphorylation of AMPKα Thr172 could be suppressed by SB202190. However we did not find such inhibitory effects after the pre-incubation with the p38 MAPK inhibitor SB 202190 (data not shown).

Finally, we evaluated the specificity of p38 MAPK involvement in telmisartan-induced eNOS phosphorylation by using dominant negative p38 MAPK overexpression by a replication-defective adenoviral vector. Overexpression of dominant negative p38 MAPK significantly inhibited telmisartan-induced eNOS phosphorylation, suggesting that the p38 MAPK pathway is predominately involved in telmisartan-induced eNOS phosphorylation. (Fig. 7F, G).

### Telmisartan-induced NO production is suppressed by p38 MAPK inhibition

**Figure 8:**
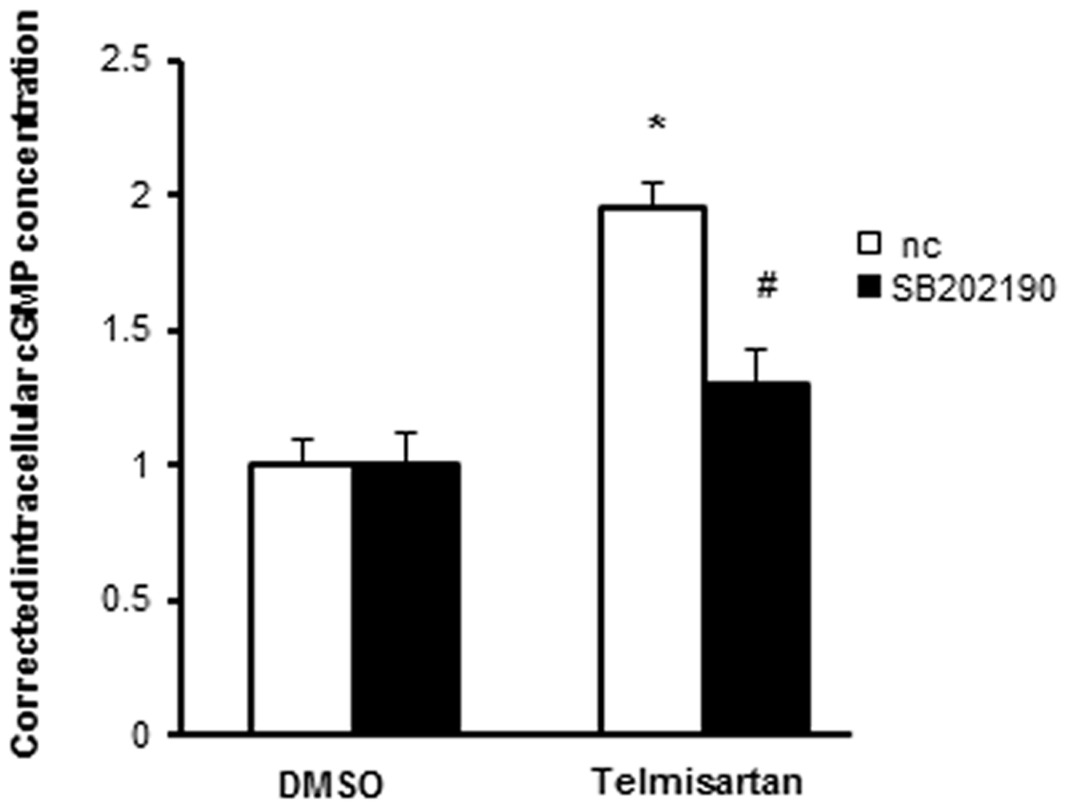
Telmisartan-induced NO production is suppressed by p38 MAPK inhibition. Intracellular cGMP concentrations were measured to evaluate NO output from HUVECs treated with telmisartan after corrected for the protein concentration. HUVECs were incubated with vehicle (DMSO) or telmisartan (10 µM) for 2 h with or without SB202190 (10 µM) pretreatment for 30 min. nc, negative control. *, p < 0.01 versus nc + DMSO; #, p < 0.01 versus nc + telmisartan.

To evaluate whether telmisartan-induced eNOS Ser1177 phosphorylation could increase NO output by activated p38 MAPK, we measured intracellular cGMP concentrations to estimate NO production by HUVECs treated with telmisartan. HUVECs were incubated with the vehicle (DMSO) or telmisartan (10 µM) for 2 h with or without 30 min of pretreatment with SB202190 (10 µM). Telmisartan increased the cGMP concentration by about 2 times compared with that in the control. However, after SB202190 pretreatment, telmisartan did not significantly increase the intracellular cGMP concentration in HUVECs (Fig. 8).

## Discussion

Telmisartan is reported to activate PPARγ, which is considered to ameliorate metabolic complications. PPARγ activation has been reported to inhibit vascular smooth muscle cell (VSMC) and fibroblast proliferation in vitro [14], [15]. Another study has shown that telmisartan is the only ARB that inhibits VSMC and cardiac fibroblast proliferation [16]. A clinical study evaluating the effects of telmisartan on the coronary plaque component and local inflammatory cytokines showed that telmisartan treatment leads to significant increases in fibrous volume and reductions in lipid volume on integrated backscatter intravascular ultrasound and significant decreases of matrix metalloproteinase (MMP)3, tumor necrosis factor-α, high-sensitive C-reactive protein and MMP9 sampled in coronary sinus [17].

In this study, we have focused on telmisartan-induced phosphorylation of eNOS and AMPK that are considered to be the dominant key players in vascular protection [18]. Telmisartan phosphorylated eNOS and AMPK significantly in HUVECs, but valsartan or irbesartan did not. Moreover, compared with valsartan, administration of telmisartan to mice significantly increased the phosphorylation of eNOS in the aortic endothelium.

We previously reported that AMPK not only protects vascular endothelium but also inhibits angiotensin II-induced VSMC proliferation [10]. Telmisartan-induced AMPK activation suggests that telmisartan has anti-arteriosclerotic functions via a different mechanism than that of ARBs in addition to the anti-arteriosclerotic functions through eNOS activation.

We investigated the intracellular signal transduction involved in telmisartan-induced eNOS phosphorylation. First, we examined the most important upstream kinase of eNOS, PI3K-Akt. Under various conditions such as pre- and post-conditioning in ischemia [19], [20], [21] and administration of insulin [22] and statins [23], [24], [25], [26], [27], [28], this pathway has been reported to play a pivotal role in cardioprotective and antiapoptotic effects. Two different kinds of PI3K inhibitor, wortmannin and LY294002, could not suppress telmisartan-induced eNOS phosphorylation, suggesting that PI3K is not necessary for this linkage. Because PKA inhibitors, KT5720 or H89, did not suppress telmisartan-induced eNOS phosphorylation, the PKA pathway was not involved either. Furthermore, we examined PPARγ and PPARβ/δ which are involved in the pleiotropic effects of telmisaratan [29], but telmisartan-induced eNOS phosphorylation was not suppressed by either GW9662 or GSK 3787, suggesting that both PPARγ and PPARβ/δ pathways were irrelevant to telmisartan-phospho-eNOS linkage.

**Figure 9:**
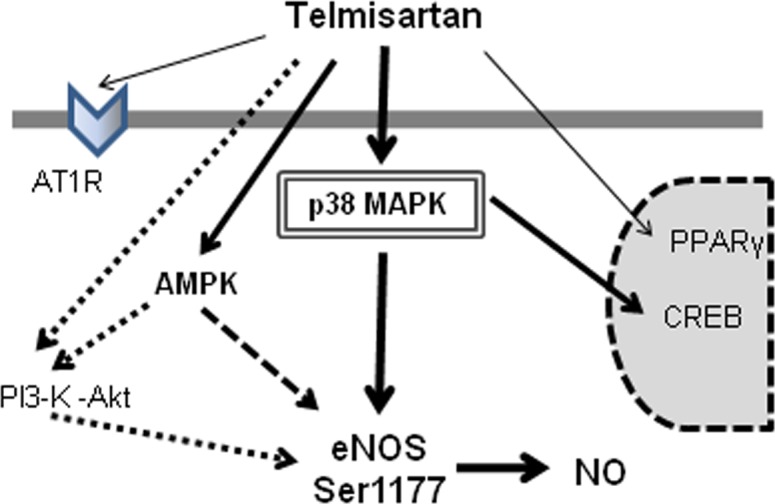
Proposed scheme for endothelial eNOS and AMPK activation by telmisartan. Telmisartan activates eNOS and increases NO production via p38 MAPK, which is independent of AT1R.

Because AMPK has been reported to be one of the major upstream kinases of eNOS, we investigated the involvement of AMPK in telmisartan-induced eNOS Ser1177 phosphorylation by using dominant negative AMPK overexpression to inhibit the AMPK pathway completely. We observed that dominant negative AMPK overexpression did not inhibit telmisartan-induced eNOS phosphorylation completely, suggesting that AMPK was not responsible for this telmisartan-eNOS cascade. Moreover, we found that telmisartan increased phosphorylation levels of CREB Ser133 and p38 MAPK Thr180/Tyr182. Inhibition of p38 MAPK by SB202190 or overexpression of dominant negative p38 MAPK significantly suppressed telmisartan-induced eNOS phosphorylation. Furthermore, pretreatment with SB202190 significantly inhibited telmisartan-induced cGMP up-regulation in HUVECs. These results revealed that telmisartan increased eNOS phosphorylation and NO output via p38 MAPK (Fig. 9).

Some previous reports have shown the association of eNOS Ser1177 phosphorylation with the p38 MAPK pathway. Kan et al. reported that 17β-estradiol increases both the expression and Ser1177 phosphorylation levels of eNOS via p38 MAPK activation [30]. They suggested that this upregulation of eNOS activity leads to inhibition of ischemic myocardial damage in rats. Anter et al. reported that black tea polyphenols activate the p38 MAPK pathway, which increases eNOS activity via Ser1177 phosphorylation and Thr495 dephosphorylation via Akt [31]. Furthermore, Grossini et al. suggested that levosimendan leads to vasodilation through NO production via p38 MAPK, ERK, and Akt pathway activation in porcine coronary endothelium [32]. p38 MAPK is activated by stimulation via cytokines and physiochemical stresses such as ultraviolet irradiation, fever, and osmotic pressure. However, the mechanism underlying telmisartan-mediated activation of the p38 MAPK pathway remains to be resolved. Telmisartan is more lipid soluble than other ARBs, and this property might be one of the reasons for p38 MAPK activation in HUVECs. Because some anti-allergy drugs are reported to affect the function of membrane-bound proteins and induce signal transduction, including calcium pathways [33], telmisartan might induce structural changes of the cell membrane and transduce phosphorylation signals. Apoptosis signal regulating kinase-1(ASK1) is one of the most pivotal upper kinase of p38 MAPK. Although functions of this kinase have been reviewed in details [34], the precise mechanisms how this kinase is activated by extracellular stress or DNA damages have been unknown. We have to investigate in the future the mechanism of telmisartan-induced p38 MAPK activation, which might include ASK1 or other upper kinases activation.

In addition to its primary role to antagonize AT1 receptor, telmisartan has unique properties which might ameliorate metabolic diseases and vascular complications via different pathway from PPARγ. We expect that a variety of clinical benefits of telmisartan concerning its unique properties would be analyzed in details and fully uncovered in the near future.

## Acknowledgments

We gratefully acknowledge the excellent technical support of Ms Asuka Ishii and Ms Marie Morita.
